# Gradient regularization of Newton method with Bregman distances

**DOI:** 10.1007/s10107-023-01943-7

**Published:** 2023-03-24

**Authors:** Nikita Doikov, Yurii Nesterov

**Affiliations:** 1 Institute of Information and Communication Technologies, Electronics and Applied Mathematics (ICTEAM), Catholic University of Louvain (UCLouvain), Louvain-la-Neuve, Belgium; 2 Center for Operations Research and Econometrics (CORE), Catholic University of Louvain (UCLouvain), Louvain-la-Neuve, Belgium

**Keywords:** Newton method, Regularization, Convex optimization, Global complexity bounds, Large-scale optimization, 49M15, 49M37, 58C15, 90C25, 90C30

## Abstract

In this paper, we propose a first second-order scheme based on arbitrary non-Euclidean norms, incorporated by Bregman distances. They are introduced directly in the Newton iterate with regularization parameter proportional to the square root of the norm of the current gradient. For the basic scheme, as applied to the composite convex optimization problem, we establish the global convergence rate of the order $O(k^{-2})$ both in terms of the functional residual and in the norm of subgradients. Our main assumption on the smooth part of the objective is Lipschitz continuity of its Hessian. For uniformly convex functions of degree three, we justify global linear rate, and for strongly convex function we prove the local superlinear rate of convergence. Our approach can be seen as a relaxation of the Cubic Regularization of the Newton method (Nesterov and Polyak in Math Program 108(1):177–205, 2006) for convex minimization problems. This relaxation preserves the convergence properties and global complexities of the Cubic Newton in convex case, while the auxiliary subproblem at each iteration is simpler. We equip our method with adaptive search procedure for choosing the regularization parameter. We propose also an accelerated scheme with convergence rate $O(k^{-3})$, where *k* is the iteration counter.

## Introduction

The classical Newton’s method is a powerful tool for solving various optimization problems and for dealing with ill-conditioning. The practical implementation of this method for solving unconstrained minimization problem $\min \nolimits _{x} f(x)$ can be written as follows:

$$\begin{aligned} x_{k + 1}= & {} x_k - \alpha _k \bigl ( \nabla ^2 f(x_k) \bigr )^{-1} \nabla f(x_k), \qquad k \ge 0, \end{aligned}$$

where $0 < \alpha _k \le 1$ is a damping parameter. However, this approach has two serious drawbacks. Firstly, the next point is not well-defined when the Hessian is a degenerate matrix. And secondly, while the method has a very fast local quadratic convergence, it is difficult to establish any *global* properties for this process. Indeed, for $\alpha _k = 1$ (the classical pure Newton method), there are known examples of problems for which the method does not converge globally [5]. The pure Newton step might not work even if the objective is strongly convex (see, e.g., Example 1.4.3 in [6]). For the damped Newton method with line search, it is possible to prove some global convergence rates. But, typically, they are worse than the rates of the classical Gradient Method [18].

A breakthrough in the second-order optimization theory was made after [19], where the Cubic Regularization of the Newton method was presented together with its global convergence properties. The main standard assumption is that the Hessian of the objective is Lipschitz continuous with some parameter $L_2 \ge 0$:

$$\begin{aligned} \Vert \nabla ^2 f(x) - \nabla ^2 f(y) \Vert\le & {} L_2 \Vert x - y\Vert , \qquad \forall x, y, \end{aligned}$$

ensuring the *global upper approximation* of our function formed by the second-order Taylor polynomial augmented by the third power of the norm. The next point is then defined as the minimum of the upper model:

1 $$\begin{aligned} x_{k + 1}= & {} \mathop {\textrm{argmin}}\limits _{y} \Bigl [ \langle \nabla f(x_k), y - x_k \rangle + \frac{1}{2} \langle \nabla ^2 f(x_k)(y - x_k), y - x_k \rangle \nonumber \\ \nonumber \\{} & {} \;\; + \; \frac{L_2}{6}\Vert y - x_k\Vert ^3 \Bigr ]. \end{aligned}$$

Initially, this idea had a full theoretical justification only for the Euclidean norm $\Vert \cdot \Vert$. In this case, the solution to the auxiliary minimization problem (1) does not have a closed form expression, but it can be found by solving a one-dimensional nonlinear equation and by using the standard factorization tools of Linear Algebra. The use of general and even variable norms with cubic regularization in second-order methods was considered recently in [11, 15], which can be useful for solving optimization problems with non-Euclidean geometry.

However, even in the Euclidean case, the presence of the cubic term in the objective makes it more difficult to use the classical gradient-type methods with their developed complexity theory. While it is possible to apply the gradient descent [2], the cubic subproblem prevents the usage of the standard accelerated and conjugate gradients methods. This drawback restricts the application of method (1) to large-scale problems.

In this paper, we show how to avoid these restrictions. Namely, we will show that it is possible to use a *quadratic regularization* of the Taylor polynomial with a properly chosen coefficient that depends only on the current iterate. In the simplest form, one iteration of our method is as follows:

2 $$\begin{aligned} x_{k + 1}= & {} x_k - \bigl ( \nabla ^2 f(x_k) + A_k I \bigr )^{-1} \nabla f(x_k), \end{aligned}$$

where

3 $$\begin{aligned} A_k= & {} \sqrt{\frac{L_2}{3} \Vert \nabla f(x_k)\Vert }. \end{aligned}$$

We see that it is very easy for implementation, since it requires only *one* matrix inversion, the very standard operation of Linear Algebra. At the same time, this subproblem is now suitable for the classical Congugate Gradient method as well.^1^

For the class of Trust Region methods as applied to unconstrained minimization problems, the trust region radius proportional to the gradient norm was proposed in [9]. The use of the gradient norm as a regularizer for the Newton method was considered in the work [20]. Then, the method has a local quadratic convergence. However, to ensure some global rate for such regularization, one need to use damping steps, which makes the rate slower.

It appears that for the optimization process (2), (3), we can establish the global convergence guarantees of the same type as for the Cubic Newton method (1). Namely, we prove the global rate of the order $O(1/k^2)$ in terms of the functional residual and in terms of the subgradient norm for the general convex functions. This is much faster than the standard *O*(1/*k*)-rate of the Gradient Method. Moreover, for the uniformly convex functions of degree three, we prove the global linear rate. For the strongly convex functions we establish a local superlinear convergence.

In this paper, we consider convex optimization problems in a general composite form. Recently, globally convergent Newton methods for nonsmooth optimization were proposed in [13]. They are based on the damping steps and regularization by the gradient norm, which is different from the rule (3).

We also work with arbitrary (possibly non-Euclidean) norms by employing the technique of Bregman distances. An alternative approach of using general norms in the cubically regularized Newton scheme was proposed in [11], that uses the adaptive regularization framework of [3].

**Contents.** The rest of the paper is organized as follows. In Sect. 2, we present the main properties of one iteration of the scheme.

We study the convergence rate of the basic process in Sect. 3. In Sect. 4, we establish convergence for the norm of the gradient. An adaptive search procedure for our method is discussed in Sect. 5.

In Sect. 6, we consider an accelerated scheme based on the iterations of the basic method and justify its global complexity of the order ${\tilde{O}}(\epsilon ^{-1/3})$ assuming Lipschitz continuity of the Hessian of the smooth part of the objective function. Section 7 contains numerical experiments. Some concluding remarks are in Sect. 8.

**Notation.** Let us fix a finite-dimensional real vector space ${\mathbb {E}}$. Our goal is to solve the following *Composite Minimization Problem*

4 $$\begin{aligned} F^* \; = \; \min \limits _{x \in \textrm{dom}\,\psi } \bigl [ F(x) \; {\mathop {=}\limits ^{\textrm{def}}}\; f(x) + \psi (x) \bigr ], \end{aligned}$$

where $\psi (\cdot )$ is a *simple* closed convex function with $\textrm{dom}\,\psi \subseteq {\mathbb {E}}$, and $f(\cdot )$ is a convex and two times continuously differentiable function.

We measure distances in ${\mathbb {E}}$ by a general norm $\Vert \cdot \Vert$. Its dual space is denoted by ${\mathbb {E}}^{*}$. It is a space of all linear functions on ${\mathbb {E}}$, for which we define the norm in the standard way:

$$\begin{aligned} \Vert g \Vert _*= & {} \max \limits _{x \in {\mathbb {E}}} \{ \; \langle g, x \rangle : \; \Vert x \Vert \le 1 \; \}, \qquad g \in {\mathbb {E}}^*. \end{aligned}$$

Using this norm, we can define an induced norm for a self-adjoint linear operator $B: {\mathbb {E}}\rightarrow {\mathbb {E}}^*$ as follows:

$$\begin{aligned} \Vert B \Vert= & {} \max \limits _{x \in {\mathbb {E}}} \{ | \langle B x, x \rangle |: \; \Vert x \Vert \le 1 \}. \end{aligned}$$

We can also define the bounds of its spectrum as the best values $\lambda _{\min }(B)$ and $\lambda _{\max }(B)$ satisfying conditions

$$\begin{aligned} \lambda _{\min }(B) \Vert x \Vert ^2\le & {} \langle B x, x \rangle \; \le \; \lambda _{\max }(B) \Vert x \Vert ^2, \quad \forall x \in {\mathbb {E}}. \end{aligned}$$

Our optimization schemes will be based on some scaling function $d(\cdot )$, which we assume to be a strongly convex function with Lipschitz-continuous gradients:

5 $$\begin{aligned}{} & {} d(y) \ge d(x) + \langle \nabla d(x), y - x \rangle + {\sigma \over 2} \Vert y - x \Vert ^2, \end{aligned}$$

6 $$\begin{aligned}{} & {} \Vert \nabla d(x) - \nabla d(y) \Vert _* \le \Vert x - y \Vert , \end{aligned}$$

where $\sigma \in (0,1]$ and the points $x, y \in \textrm{dom}\,\psi$ are arbitrary. For twice-differentiable scaling functions, this condition can be characterized by the following bounds on the Hessian:

$$\begin{aligned} \sigma \Vert h\Vert ^2\le & {} \langle \nabla ^2 d(x)h, h \rangle \;\; \le \;\; \Vert h\Vert ^2, \qquad \forall x \in \textrm{dom}\,\psi , \; h \in {\mathbb {E}}. \end{aligned}$$

Using this function, we define the following *Bregman distance*:

7 $$\begin{aligned} \rho (x,y)= & {} \beta _d(x,y) \;\; {\mathop {=}\limits ^{\textrm{def}}}\;\; d(y) - d(x) - \langle \nabla d(x), y - x \rangle , \quad x, y \in \textrm{dom}\,\psi . \nonumber \\ \end{aligned}$$

We will employ this object to regularize the second-order model of the objective.

The standard condition for the smooth part of the objective function in problem (4) is Lipschitz continuity of the Hessians:

8 $$\begin{aligned} \Vert \nabla ^2 f(x) - \nabla ^2 f(y) \Vert \;\; \le \;\; L_2 \Vert x - y \Vert , \qquad \forall x, y \in \textrm{dom}\,\psi , \end{aligned}$$

that we always assume to be satisfied. This inequality has the following consequences, which are valid for all $x,y \in \textrm{dom}\,\psi$:

9 $$\begin{aligned} \Vert \nabla f(y) - \nabla f(x) - \nabla ^2 f(x)(y-x) \Vert _* \;\; \le \;\; {1 \over 2}L_2 \Vert y - x \Vert ^2, \end{aligned}$$

and

10 $$\begin{aligned}{} & {} |f(y) - f(x) - \langle \nabla f(x), y - x \rangle + {1 \over 2}\langle \nabla ^2 f(x)(y-x), y-x \rangle | \nonumber \\{} & {} \quad \quad \quad \le \;\; \frac{1}{6} L_2 \Vert y - x \Vert ^3. \end{aligned}$$

## Gradient regularization

Our main iteration at some point ${\bar{x}} \in \textrm{dom}\,\psi$ with a step-size $A>0$ is defined as follows:

11 $$\begin{aligned}{} & {} T_A({\bar{x}}) \;\; {\mathop {=}\limits ^{\textrm{def}}}\;\; \mathop {\textrm{argmin}}\limits _{y \in \textrm{dom}\,\psi } \Big [ \; M_A({\bar{x}}, y) {\mathop {=}\limits ^{\textrm{def}}}f({\bar{x}}) + \langle \nabla f({\bar{x}}), y - {\bar{x}} \rangle \nonumber \\{} & {} \quad \quad + {1 \over 2}\langle \nabla ^2 f({\bar{x}})(y - {\bar{x}}), y - {\bar{x}} \rangle + A \rho ({\bar{x}}, y) + \psi (y) \; \Big ]. \end{aligned}$$

This is minimization of a convex quadratic function augmented by Bregman distance and the composite part. Our main structural assumption is that both $\rho (\bar{x}, \cdot )$ and $\psi (\cdot )$ are *simple*, meaning that problem (11) is efficiently solvable.

The use of the general scaling function $d( \cdot )$ can be beneficial in practice for solving problems with some specific non-Euclidean geometry.

### Example 1

Let $\psi (x) \equiv 0$ and the scaling function is $d(x):= \frac{1}{2}\langle B x, x \rangle$ for a fixed positive definite self-adjoint operator $B = B^{*} \succ 0$. Then,

$$\begin{aligned} \rho (\bar{x}, y)= & {} \frac{1}{2} \langle B(y - \bar{x}), y - \bar{x} \rangle , \end{aligned}$$

and one iteration (11) can be written in an explicit form, as follows:

$$\begin{aligned} T_A(\bar{x})= & {} \bar{x} - \bigl ( \nabla ^2 f(\bar{x}) + A B \bigr )^{-1} \nabla f(\bar{x}). \end{aligned}$$

### Example 2

Consider the unconstrained minimization problem $\min _{x \in {\mathbb {R}}^n} f(x)$, with

$$\begin{aligned} f(x)= & {} g(Cx), \qquad C \in {\mathbb {R}}^{m \times n}, \end{aligned}$$

where $g: {\mathbb {R}}^m \rightarrow {\mathbb {R}}$ is a convex smooth function. Let us fix the standard Euclidean norm $\Vert \cdot \Vert _2$ in ${\mathbb {R}}^m$ and assume that the Hessian of *g* is Lipschitz continuous w.r.t. this norm with constant $L_g$. Then, if we use the standard Euclidean norm $\Vert \cdot \Vert _2$ for our primal space ${\mathbb {R}}^n$, the corresponding Lipschitz constant of $\nabla ^2 f(\cdot )$ is

$$\begin{aligned} L_f= & {} \Vert C\Vert ^3 L_g. \end{aligned}$$

At the same time, using the following scaled norm $\Vert x \Vert := \langle Bx, x\rangle ^{1/2}$, $x \in {\mathbb {R}}^n$ with matrix $B = C^T C$ (assuming $B \succ 0$, so the rows of *C* have a full rank) and the scaling function from the previous example, we have

$$\begin{aligned} L_f= & {} L_g, \end{aligned}$$

which is much better.

### Example 3

Let $\psi (\cdot )$ be $\{0, +\infty \}$-indicator of the standard simplex

$$\begin{aligned} \varDelta _n {\mathop {=}\limits ^{\textrm{def}}}\left\{ x \in {\mathbb {R}}^n_+ \;: \; \sum _{i = 1}^n x^{(i)} = 1 \right\} . \end{aligned}$$

Thus, problem (4) is to minimize a smooth convex function over this set:

$$\begin{aligned} \min \limits _{x \in \varDelta _n} f(x). \end{aligned}$$

One of the most suitable choices of the norm for this problem is $\ell _1$-norm [1], defined as $\Vert x \Vert _1 {\mathop {=}\limits ^{\textrm{def}}}\sum _{i = 1}^n |x^{(i)}|$ for $x \in {\mathbb {R}}^n$. The Lipschitz constant w.r.t. this norm is smaller than that one measured in $\ell _2$-norm. Let us fix some $\delta > 0$, and use the following scaling function,

$$\begin{aligned} d(x):= & {} \delta \sum \limits _{i = 1}^n (x^{(i)} + \delta ) \ln (x^{(i)} + \delta ). \end{aligned}$$

We have, for any $h \in {\mathbb {R}}^n$ and $x \in \varDelta _n$:

$$\begin{aligned} \langle \nabla ^2 d(x)h, h \rangle= & {} \delta \sum \limits _{i = 1}^n \frac{( h^{(i)} )^2 }{ x^{(i)} + \delta } \;\; \le \;\; \Vert h\Vert _2^2 \;\; \le \;\; \Vert h\Vert _1^2. \end{aligned}$$

And, by Cauchy-Schwarz inequality, it holds

$$\begin{aligned} \Vert h\Vert _1= & {} \sum \limits _{i = 1}^n \frac{| h^{(i)} | \sqrt{ x^{(i)} + \delta } }{\sqrt{ x^{(i)} + \delta } } \;\; \le \;\; \biggl (\sum \limits _{i = 1}^n \frac{(h^{(i)})^2}{x^{(i)} + \delta } \biggr )^{1/2} \bigl ( 1 + n\delta \bigr )^{1/2}. \end{aligned}$$

Hence,

$$\begin{aligned} \langle \nabla ^2 d(x)h, h \rangle\ge & {} \frac{\delta }{1 + n \delta } \Vert h\Vert _1^2. \end{aligned}$$

and conditions (5), (6) are satisfied with $\sigma = \frac{\delta }{1 + n \delta } = \frac{1}{1/\delta + n}$.

In general, the solution to this problem $T = T_A({\bar{x}})$ is characterized by the following variational principle (see, e.g. [18]):

12 $$\begin{aligned}{} & {} \langle \nabla f({\bar{x}}) + \nabla ^2f({\bar{x}})(T-{\bar{x}}) + A (\nabla d(T) - \nabla d({\bar{x}})), y - T \rangle \nonumber \\{} & {} \quad + \; \psi (y)\; \ge \; \psi (T), \quad \forall y \in \textrm{dom}\,\psi . \end{aligned}$$

Thus, defining $\psi '(T) = - \nabla f({\bar{x}}) - \nabla ^2f({\bar{x}})(T-{\bar{x}}) - A (\nabla d(T) - \nabla d({\bar{x}}))$, we see that $\psi '(T) \in \partial \psi (T)$. Consequently,

13 $$\begin{aligned} F'(T)= & {} \nabla f(T) + \psi '(T)\nonumber \\= & {} \; \nabla f(T) - \nabla f({\bar{x}}) - \nabla ^2f({\bar{x}})(T-{\bar{x}})\nonumber \\{} & {} - A (\nabla d(T) - \nabla d({\bar{x}})) \; \in \; \partial F(T). \end{aligned}$$

Note that this is a very special way of selecting subgradient of a possibly nonsmooth function $F(\cdot )$, which allows $\Vert F'(T) \Vert _*$ approach zero.

Denote $M_A({\bar{x}}) = M_A({\bar{x}}, T_A({\bar{x}})) \le M_A({\bar{x}}, {\bar{x}}) = F({\bar{x}})$. Let us prove the following important fact, that uses convexity of the original problem (4).

### Lemma 1

For all $y \in \textrm{dom}\,\psi$ and $T = T_A({\bar{x}})$, we have

14 $$\begin{aligned} M_A({\bar{x}}, y)\ge & {} M_A({\bar{x}}) + {1 \over 2}\langle \nabla ^2 f({\bar{x}}) (y - T), y - T \rangle + {1 \over 2}\sigma A\Vert y - T \Vert ^2. \end{aligned}$$

Moreover,

15 $$\begin{aligned} \Vert T_A({\bar{x}}) - {\bar{x}} \Vert\le & {} {1 \over \sigma A} \Vert F'({\bar{x}}) \Vert _*, \end{aligned}$$

where $F'({\bar{x}}) = \nabla f({\bar{x}}) + \psi '({\bar{x}})$ and $\psi '({\bar{x}})$ is an arbitrary element of $\partial \psi ({\bar{x}})$.

### Proof

For optimization problem in (11), define the scaling function

$$\begin{aligned} \xi (x)= & {} {1 \over 2}\langle \nabla ^2f({\bar{x}}) x, x \rangle + A d(x). \end{aligned}$$

Note that the objective function in this problem is strongly convex relatively to $\xi (\cdot )$ with constant one. Therefore, for any $y \in \textrm{dom}\,\psi$,

$$\begin{aligned} M_A({\bar{x}}, y) - M_A({\bar{x}})&\ge \beta _{\xi }(T,y) \; = \; {1 \over 2}\langle \nabla ^2 f({\bar{x}}) (y - T), y - T \rangle + A \beta _d(T,y) \\&{\mathop {\ge }\limits ^{(15)}} {1 \over 2}\langle \nabla ^2 f({\bar{x}}) (y - T), y - T \rangle + {1 \over 2}\sigma A \Vert y - T \Vert ^2. \end{aligned}$$

In order to prove (15), note that

$$\begin{aligned} M_A({\bar{x}})\ge & {} F({\bar{x}}) + \min \limits _{y \in \textrm{dom}\,\psi } \Big [ \langle F'({\bar{x}}), y - {\bar{x}} \rangle + {1 \over 2}\sigma A \Vert y - {\bar{x}} \Vert ^2 \Big ]\\\ge & {} F({\bar{x}}) + \min \limits _{y \in {\mathbb {E}}} \Big [ \langle F'({\bar{x}}), y - {\bar{x}} \rangle + {1 \over 2}\sigma A \Vert y - {\bar{x}} \Vert ^2 \Big ]\\= & {} F({\bar{x}}) - {1 \over 2 \sigma A} \Vert F'({\bar{x}}) \Vert _*^2. \end{aligned}$$

Since $M_A({\bar{x}}, {\bar{x}}) = F({\bar{x}})$, we get (15) from (14) with $y = {\bar{x}}$. $\square$

In what follows, the parameter *A* in the optimization problem (11) is chosen as

16 $$\begin{aligned} A \; = \; A_H({\bar{x}}) \; = \; {1 \over \sigma } \sqrt{{H \over 3 } \Vert F'({\bar{x}}) \Vert _*}, \end{aligned}$$

where $H > 0$ is an estimate of the Lipschitz constant $L_2$ in (8). This choice is explained by the following result.

### Corollary 1

For $A = A_H({\bar{x}})$, we have

17 $$\begin{aligned} H \Vert T_A({\bar{x}}) - {\bar{x}} \Vert\le & {} 3 \sigma A. \end{aligned}$$

### Proof

Indeed, this is a simple consequence of inequality (15) and definition (11). $\square$

Let us relate the optimal value of the auxiliary problem (11) with the cubic over-approximation (10).

### Lemma 2

Let $A = A_H({\bar{x}})$ and $T = T_A({\bar{x}})$. Assume that for some $H > 0$ the following condition is satisfied:

18 $$\begin{aligned} f(T)\le & {} f({\bar{x}}) + \langle \nabla f({\bar{x}}), T - {\bar{x}} \rangle \nonumber \\{} & {} + {1 \over 2}\langle \nabla ^2f({\bar{x}})(T - {\bar{x}}), T - {\bar{x}} \rangle + {H \Vert T - {\bar{x}} \Vert ^3 \over 6} \end{aligned}$$

(clearly, it holds for $H \ge L_2$, where $L_2$ is the Lipschitz constant of the Hessian). Then

19 $$\begin{aligned} F({\bar{x}}) - F(T)\ge & {} {1 \over 2}\langle \nabla ^2 f({\bar{x}}) (T - {\bar{x}}), T - {\bar{x}} \rangle + {1 \over 2}\sigma A\Vert T - {\bar{x}} \Vert ^2. \end{aligned}$$

### Proof

Indeed,

$$\begin{aligned} f(T)&{\mathop {\le }\limits ^{(18)}}&M_A({\bar{x}}) - A \rho ({\bar{x}},T) -\psi (T) +{H \over 6} \Vert T - {\bar{x}} \Vert ^3\\&{\mathop {\le }\limits ^{(5)}}&M_A({\bar{x}}) -\psi (T) +{H \over 6} \Vert T - {\bar{x}} \Vert ^3 - {1 \over 2}\sigma A \Vert T - {\bar{x}} \Vert ^2\\&{\mathop {\le }\limits ^{(17)}}&M_A({\bar{x}}) -\psi (T). \end{aligned}$$

Thus, $F(T) \le M_A({\bar{x}})$ and (19) follows from (14) with $y = {\bar{x}}$. $\square$

Finally, we need to estimate the norm of subgradient at the new point.

### Lemma 3

Let the Hessian be Lipschitz continuous with constant $L_2$. Fix arbitrary $H > 0$. Let $A = A_H({\bar{x}})$ and $T = T_A({\bar{x}})$. Then

20 $$\begin{aligned} \Vert F'(T) \Vert _*\le & {} \sigma A \left( \sigma ^{-1} + {3 L_2 \over 2 H} \right) \Vert T - {\bar{x}} \Vert \; \le \; c \Vert F'({\bar{x}} ) \Vert _*, \end{aligned}$$

where

$$\begin{aligned} c {\mathop {=}\limits ^{\textrm{def}}}\sigma ^{-1} + {3 L_2 \over 2 H}. \end{aligned}$$

### Proof

Indeed,

$$\begin{aligned} \Vert F'(T) \Vert _*&{\mathop {=}\limits ^{(13)}} \Vert \nabla f(T) - \nabla f({\bar{x}}) {-} \nabla ^2f({\bar{x}})(T{-}{\bar{x}}) {-} A (\nabla d(T){ -} \nabla d({\bar{x}})) \Vert _*\\&{\mathop {\le }\limits ^{(9)}} {1 \over 2}L_2 \Vert T {-} {\bar{x}} \Vert ^2 {+} A \Vert \nabla d(T) {-} \nabla d({\bar{x}}) \Vert _* \; {\mathop {\le }\limits ^{(6)}} \; {1 \over 2}L_2 \Vert T {-} {\bar{x}} \Vert ^2 {+} A \Vert T {-} {\bar{x}} \Vert _*\\&{\mathop {\le }\limits ^{(17)}} A \left( 1 {+} {3 \sigma L_2 \over 2 H} \right) \Vert T {-} {\bar{x}} \Vert . \end{aligned}$$

This is the first inequality in (20). For the second one, we can continue as follows:

$$\begin{aligned} \Vert F'(T) \Vert _*&{\mathop {\le }\limits ^{(17)}}&\left( 1 + {3 \sigma L_2 \over 2 H} \right) \cdot {3 \sigma A^2 \over H} \; {\mathop {=}\limits ^{(17)}} \; c \Vert F'({\bar{x}} ) \Vert _*. \end{aligned}$$

$$\square $$

Now we can prove the main theorem of this section.

### Theorem 1

Let the Hessian be Lipschitz continuous with constant $L_2$. Fix arbitrary $H > 0$. Let $A = A_H({\bar{x}})$ and $T = T_A({\bar{x}})$. If for this point relation (18) is valid, then

21 $$\begin{aligned} F({\bar{x}}) - F(T)\ge & {} {1 \over 2 c^2 } \sqrt{3 \over H} \cdot { \Vert F'(T) \Vert _*^2 \over \Vert F'({\bar{x}}) \Vert _*^{1/2}}. \end{aligned}$$

### Proof

We only need to insert in (19) the first inequality of (20) and definition (16). $\square$

## Properties of the minimization process

In this section, we propose an iterative scheme based on the gradient regularization of the Newton steps. Note that the choice of the regularization parameter (16) depends solely on the *current gradient norm* and it can be easily computed at each iteration. Then, we do one regularized Newton step defined by (11). According to Theorem 1, repeating this process would result in monotone decrease of the objective.

First, we prove global convergence for the function value. In the next section, we also prove the convergence in terms of the gradient norm. Thus, small gradient norm can serve as a stopping criteria for our scheme.

Let us analyze the following algorithm with a fixed value of parameter *H*.22Let us introduce the distance to the initial level set:

$$\begin{aligned} D= & {} \sup \limits _{x \in \textrm{dom}\,\psi } \{ \Vert x - x^* \Vert : \; F(x) \le F(x_0) \}, \end{aligned}$$

which we assume to be bounded: $D < +\infty$. We can prove the following convergence rate for method (22).

### Theorem 2

Let the Hessian be Lipschitz continuous with constant $L_2$. Let $H \ge L_2$ and $F(x_k) - F^* \ge \epsilon$ for some $k \ge 0$. Then,

23 $$\begin{aligned} {1 \over [F(x_k) - F^*]^{1/2}}\ge & {} {1 \over [F(x_0) - F^*]^{1/2}} \nonumber \\{} & {} \; + \; {1 \over 4 c^2} \sqrt{3 \over H D^3} \left( k - \ln {(F(x_0) - F^*) \Vert F'(x_0) \Vert _*^{1/2}D^{1/2} \over \epsilon ^{3/2}} \right) .\nonumber \\ \end{aligned}$$

### Proof

Denote $F_k = F(x_k) - F(x^*)$ and $g_k = \Vert F'(x_k) \Vert _*$. Thus, $F_k \le D g_k$. Note that

$$\begin{aligned} {1 \over F_{k+1}^{1/2}} - {1 \over F_k^{1/2}}= & {} { F_{k}^{1/2} - F_{k+1}^{1/2} \over F_{k}^{1/2} F_{k+1}^{1/2}} \; = \; {F_k - F_{k+1} \over F_{k}^{1/2} F_{k+1}^{1/2} (F_{k}^{1/2} + F_{k+1}^{1/2})} \; \ge \; {F_k - F_{k+1} \over 2 F_{k} F_{k+1}^{1/2}}. \end{aligned}$$

Since for all $k \ge 1$, the subgradients of $\psi (\cdot )$ are defined by the rule (13), we can use the results of Sect. 2. We can continue as follows:

$$\begin{aligned} {1 \over F_{k{+}1}^{1/2}} {-} {1 \over F_k^{1/2}}&{\mathop {\ge }\limits ^{(21)}}&{\sqrt{3} g_{k{+}1}^2 \over 4 \sqrt{H} c^2 g_k^{1/2} F_{k} F_{k{+}1}^{1/2}} \; {\ge } \; {\sqrt{3}g_{k{+}1}^{1/2} F_{k{+}1} \over 4 \sqrt{H} c^2 g_k^{1/2} F_{k} D^{3/2}}\; {=} \; {g_{k{+}1}^{1/2} F_{k{+}1} \over 4c^2 g_k^{1/2} F_{k}} \sqrt{3 \over HD^3}. \end{aligned}$$

Summing up these bounds and using the inequality of arithmetic and geometric means, we get

24 $$\begin{aligned} {1 \over F_k^{1/2}} - {1 \over F_0^{1/2}}\ge & {} {1 \over 4 c^2} \sqrt{3 \over HD^3} \sum \limits _{i=0}^{k-1} {F_{i+1} g_{i+1}^{1/2} \over F_i g_i^{1/2}} \; \ge \; {k \over 4c^2} \sqrt{3 \over HD^3} \left( {F_{k} g_{k}^{1/2} \over F_0 g_0^{1/2}}\right) ^{1/k}\nonumber \\\ge & {} {k \over 4c^2} \sqrt{3 \over HD^3} \left( {\epsilon ^{3/2} \over F_0 g_0^{1/2}D^{1/2}}\right) ^{1/k}. \end{aligned}$$

Since

$$\begin{aligned} \left( {\epsilon ^{3/2} \over F_0 g_0^{1/2}D^{1/2}}\right) ^{1/k}= & {} \exp \Big ( - {1 \over k} \ln {F_0 g_0^{1/2}D^{1/2} \over \epsilon ^{3/2}} \Big ) \; \ge 1 - {1 \over k} \ln {F_0 g_0^{1/2}D^{1/2} \over \epsilon ^{3/2}}, \end{aligned}$$

we obtain inequality (23). $\square$

### Corollary 2

The second condition of Theorem 2 can be valid only for

25 $$\begin{aligned} k\le & {} 4 c^2 \sqrt{ H D^3 \over 3 \epsilon } + \ln {(F(x_0) - F^*) \Vert F'(x_0) \Vert _*^{1/2}D^{1/2} \over \epsilon ^{3/2}}. \end{aligned}$$

### Remark 1

Note that up to the additive logarithmic term, the iteration complexity (25) corresponds to that one of the Cubically regularized Newton method as applied to convex functions [19] in the Euclidean case. However, iterations of our method (22) are easier to implement, and it is also possible to use an arbitrary scaling function $d(\cdot )$.

### Remark 2

The right-hand side of inequality (25) can be used for defining the optimal value of parameter *H*. Indeed, it can be chosen as a minimizer of the following function:

$$\begin{aligned} 2 \ln (2H\sigma ^{-1} + 3L_2) - {3 \over 2} \ln H. \end{aligned}$$

This gives us

26 $$\begin{aligned} H_*= & {} {9 \over 2} L_2 \sigma . \end{aligned}$$

In this case,

27 $$\begin{aligned} 4c^2\sqrt{ H_* D^3 \over 3 \epsilon }= & {} {64 \over 9 \sigma } \sqrt{ 3 L_2 D^3 \over 2 \epsilon \sigma } \; < \; 8.71 \sqrt{ L_2 D^3 \over \epsilon \sigma ^3}. \end{aligned}$$

Let us estimate now the performance of method (22) on uniformly convex functions. Consider the case when function $F(\cdot )$ is uniformly convex of degree three:

28 $$\begin{aligned} F(y)\ge & {} F(x) + \langle F'(x), y - x \rangle + {\sigma _3 \over 3} \Vert y - x \Vert ^3, \quad x, y \in \textrm{dom}\,\psi . \end{aligned}$$

For the composite $F(\cdot )$, this property can be ensured either by its smooth component $f(\cdot )$, or by the general component $\psi (\cdot )$. In the latter case, it is not necessary to coordinate this assumption with the smoothness condition (8).

In our analysis, we need the following straightforward consequence of definition (28):

29 $$\begin{aligned} F(x) - F^*\le & {} {2 \over 3 \sqrt{\sigma _3}} \Vert F'(x) \Vert _*^{3/2}, \quad x \in \textrm{dom}\,\psi . \end{aligned}$$

### Theorem 3

Let the Hessian be Lipschitz continuous with constant $L_2$. Let $F(\cdot )$ satisfies condition (28). If $H \ge L_2$, then for all $k \ge 0$ we have

30 $$\begin{aligned} F(x_k) - F^*\le & {} D\Vert F'(x_0) \Vert _* \cdot \exp \left( - {k \ln (1+S) \over c^{1/2} + {1 \over 2}\ln (1+S)} \right) , \end{aligned}$$

where $$S = {3 \sqrt{3} \over 4 c^{3/2} } \sqrt{\sigma _3 \over H}$$.

### Proof

As in the proof of Theorem 2, denote $F_k = F(x_k) - F^*$ and $g_k = \Vert F'(x_k) \Vert _*$. Then, we have

$$\begin{aligned} \ln {1 \over F_{k+1}} - \ln {1 \over F_k}&= \ln \left( 1 + {F_k - F_{k+1} \over F_{k+1}} \right) \; {\mathop {\ge }\limits ^{(21)}} \; \ln \left( 1 + {\sqrt{3} g_{k+1}^2 \over 2 \sqrt{H} c^2 g_k^{1/2} F_{k+1}} \right) \\&{\mathop {\ge }\limits ^{(29)}} \ln \left( 1 + {3 \over 4c^2} \sqrt{3 \sigma _3 \over H} \cdot {g_{k+1}^{1/2} \over g_k^{1/2}} \right) \; = \; \ln \left( 1 + S \cdot \sqrt{g_{k+1} \over c g_k } \right) , \end{aligned}$$

where $S = {3 \over 4c^{3/2}} \sqrt{3 \sigma _3 \over H}$. Denote $\tau _k = \sqrt{g_{k+1} \over c g_k } {\mathop {\le }\limits ^{(20)}} 1$. Since $\ln (\cdot )$ is a concave function, we have $\ln (1+S \tau _k) \ge \tau _k \ln (1+S)$. Hence,

$$\begin{aligned} \xi _k {\mathop {=}\limits ^{\textrm{def}}}\ln {g_0 D \over F_k} \; \ge \; \ln {F_0 \over F_k}\ge & {} \ln (1+S) \sum \limits _{i=0}^{k-1} \tau _i \; \ge \; {k \over c^{1/2}} \ln (1+S) \left( \prod \limits _{i=0}^{k-1} {g_{i+1}^{1/2} \over g_i^{1/2}}\right) ^{1/k} \\= & {} {k \over c^{1/2}} \ln (1+S) \left( {g_k \over g_0}\right) ^{1/(2k)}. \end{aligned}$$

Note that $\left( {g_k \over g_0}\right) ^{1/(2k)} = \exp \left( - {1 \over 2k} \ln {g_0 \over g_k} \right) \ge 1 + {1 \over 2k} \ln {g_k \over g_0} \ge 1 + {1 \over 2k} \ln {F_k \over g_0 D} = 1 - {1 \over 2k} \xi _k$. Thus,

$$\begin{aligned} \xi _k\ge & {} {k \ln (1+S) \over c^{1/2} + {1 \over 2}\ln (1+S)}, \end{aligned}$$

and this is inequality (30). $\square$

### Remark 3

in accordance to the estimate (30), the highest rate of convergence corresponds to the maximal value of *S*. This means that we need to minimize the factor $c^{3/2} H^{1/2}$ in *H*. The optimal value is given by $H_{\#} = {3 \sigma }L_2$. In this case,

31 $$\begin{aligned} S= & {} {\sigma } \sqrt{\sigma _3 \over 6 L_2} \; > \; 0.4 \sigma \sqrt{\sigma _3 \over L_2}. \end{aligned}$$

Note that this condition number also corresponds to the global convergence of the Cubically regularized Newton method [8].

Finally, let us prove a superlinear rate of local convergence for scheme (22).

### Theorem 4

Let the Hessian be Lipschitz continuous with constant $L_2$. Let function $f(\cdot )$ be strongly convex on $\textrm{dom}\,\psi$ with parameter $\mu > 0$. If $H \ge L_2$, then for all $k \ge 0$ we have

32 $$\begin{aligned} \Vert F'(x_{k+1} ) \Vert _*\le & {} {2 c\over \mu } \sqrt{H \over 3} \Vert F'(x_k) \Vert _*^{3/2}. \end{aligned}$$

### Proof

Indeed, for any $k \ge 0$ we have

$$\begin{aligned} {\mu \over 2} \Vert x_{k+1} - x_k \Vert ^2&\le {1 \over 2}\langle \nabla ^2f(x_k)(x_{k+1} - x_k), x_{k+1} - x_k \rangle \\&{\mathop {\le }\limits ^{(19)}} F(x_k) - F(x_{k+1}) \; \le \; \Vert F'(x_k) \Vert _* \Vert x_k - x_{k+1} \Vert . \end{aligned}$$

Therefore,

$$\begin{aligned} \Vert F'(x_{k+1}) \Vert _*&{\mathop {\le }\limits ^{(20)}} \sigma c A_k \Vert x_{k+1} - x_k \Vert \; \le \; {2 \sigma c \over \mu } A_k \Vert F'(x_k) \Vert _*\\&{\mathop {=}\limits ^{(16)}} {2 c \over \mu } \sqrt{H \over 3} \Vert F'(x_k) \Vert _*^{3/2}. \end{aligned}$$

$$\square $$

Thus, the region of superlinear convergence of method (22) is as follows:

33 $$\begin{aligned} {{{\mathcal {R}}}}_Q {\mathop {=}\limits ^{\textrm{def}}}\left\{ x \in \textrm{dom}\,\psi :\; \Vert F'(x) \Vert _* \le {3 \mu ^2 \over 4 H c^2} \right\} . \end{aligned}$$

Note that outside this region, the constant of strong convexity of the objective function in problem (11) with $A = A_H(x)$ satisfies the following lower bound:

34 $$\begin{aligned} \sigma A_H(x)\ge & {} {\mu \over 2c}, \quad x \not \in \mathcal{R}_Q. \end{aligned}$$

## Estimating the norm of the gradient

Let us estimate the efficiency of method (22) in decreasing the norm of gradients. For that, we are going to derive an upper bound for the number of steps *N* of method (22), for which we still have

35 $$\begin{aligned} \Vert F'(x_k) \Vert _* \ge \delta > 0, \quad 0 \le k \le N. \end{aligned}$$

We will see that global complexities of our method for minimizing the gradient norm in convex case are the same as that one of the basic Cubic Newton [10].

In this section, we use notation of Sect. 3:

$$\begin{aligned} F_k= & {} F(x_k) - F^*, \quad g_k \; = \; \Vert F'(x_k) \Vert _*. \end{aligned}$$

Firstly, consider the case when the smooth component $f(\cdot )$ in the objective function of problem (4) satisfies condition (8). Then

36 $$\begin{aligned} F_k - F_{k+1}&{\mathop {\ge }\limits ^{(21)}}&\kappa {g_{k+1}^2 \over g_k^{1/2}}, \quad \kappa {\mathop {=}\limits ^{\textrm{def}}}{1 \over 2c^2} \sqrt{3 \over H}. \end{aligned}$$

It is convenient to assume that the number of iteration *N* of the method is a multiple of three:

37 $$\begin{aligned} N = 3m, \quad m \ge 1. \end{aligned}$$

Then for the last *m* iterations of the scheme we have

38 $$\begin{aligned} F_{2m}\ge & {} F_{2m} - F_{3m} \; \ge \; \kappa \sum \limits _{i=0}^{m-1} {g_{2m+i+1}^2 \over g_{2m+i}^{1/2}} \; {\mathop {\ge }\limits ^{(35)}} \; \kappa \delta ^{3/2} \sum \limits _{i=0}^{m-1} {g_{2m+i+1}^{1/2} \over g_{2m+i}^{1/2}}\nonumber \\\ge & {} \kappa m \delta ^{3/2} \left( {g_{3m}^{1/2} \over g_{2m}^{1/2}}\right) ^{1/m} \; {\mathop {\ge }\limits ^{(35)}} \; \kappa m \delta ^{3/2} \left( {\delta ^{1/2} \over g_{2m}^{1/2}}\right) ^{1/m}. \end{aligned}$$

At the same time, for the first 2*m* iterations we obtain

39 $$\begin{aligned} {1 \over F_{2m}^{1/2}} - {1 \over F_0^{1/2}}&{\mathop {\ge }\limits ^{(24)}} {2 m \over 4 c^2} \sqrt{3 \over H D^3} \left( {F_{2m}g_{2m}^{1/2} \over F_0 g_0^{1/2}} \right) ^{1/(2m)} \nonumber \\&= \kappa m D^{-3/2}\left( {F_{2m}g_{2m}^{1/2} \over F_0 g_0^{1/2}} \right) ^{1/(2m)}. \end{aligned}$$

Therefore,

40 $$\begin{aligned} \biggl ( \frac{1}{F_{2m}^{1/2}} - \frac{1}{F_0^{1/2}} \biggr )^{\!2}\overset{(39)}{\ge } & {} (km)^2 D^{-3} \biggl ( \frac{F_{2m} g_{2m}^{1/2}}{F_0 g_0^{1/2}} \biggr )^{1 / m}. \end{aligned}$$

Note that the power of $g_{2m}$ in the last term is equal to that one of $\frac{1}{g_{2m}}$ in (38). This explains our choice 2*m* for the length of the first stage.

Hence, using both inequalities (38) and (40), we obtain the following:

$$\begin{aligned} 1\ge & {} \left( 1 - \sqrt{F_{2m} \over F_0} \right) ^2 \; = \; \left( {1 \over F_{2m}^{1/2}} - {1 \over F_0^{1/2}} \right) ^2 \cdot F_{2m} \; \ge \; \left( { \kappa m \delta ^{1/2} \over D} \right) ^3 \left( {F_{2m} \delta ^{1/2} \over F_0 g_0^{1/2}} \right) ^{1/m} \end{aligned}$$

Note that $g_{2m} {\mathop {\le }\limits ^{(20)}} c^{2m} g_0$. Therefore,

$$\begin{aligned} F_{2m}&{\mathop {\ge }\limits ^{(38)}}&\kappa m \delta ^{3/2} \left( {\delta ^{1/2} \over c^m g_0^{1/2}}\right) ^{1/m}, \end{aligned}$$

and we obtain

$$\begin{aligned} 1\ge & {} \left( { \kappa m \delta ^{1/2} \over D} \right) ^3 \left( { \kappa m \delta ^{2} \over c F_0 g_0^{1/2}} \cdot \left( {\delta ^{1/2} \over g_0^{1/2}}\right) ^{1/m}\right) ^{1/m} \\\ge & {} \left( {\kappa m \delta ^{1/2} \over D} \right) ^{3 + {1 \over m}} \left( {\delta ^{1/2} \over g_0^{1/2}}\right) ^{ (3 + {1 \over m}) {1 \over m}} \Big ( c \Big )^{-{1 \over m}}. \end{aligned}$$

Thus, we can prove the following theorem.

### Theorem 5

Let the Hessian be Lipschitz continuous with constant $L_2$. Fix $H \ge L_2$ and some $\delta >0$. Then, the number of iterations of method (22) to reach small norm of the gradient $\Vert F'(x_N) \Vert _{*} \le \delta$ satisfies the following bound:

41 $$\begin{aligned} N\le & {} 2 c^2 \sqrt{3 H D^2 \over \delta } + {3 \over 2} \ln {g_0 \over \delta } + \ln c. \end{aligned}$$

### Proof

Indeed,

$$\begin{aligned} 1\ge & {} {\kappa m \delta ^{1/2} \over D} \Big ( {\delta \over g_0} \Big )^{1 \over 2m} \Big ( c \Big )^{-{1 \over 3m+1}} \; = \; {\kappa m \delta ^{1/2} \over D} \exp \left( - {1 \over 2m} \ln \left[ {g_0 \over \delta } \Big ( c \Big )^{{2 m \over 3 m+1}} \right] \right) \\\ge & {} {\kappa \delta ^{1/2} \over D} \left( m - {1 \over 2}\ln {g_0 \over \delta } - {m \over 3m+1} \ln c \right) \; \ge \; {\kappa \delta ^{1/2} \over D} \left( m - {1 \over 2}\ln {g_0 \over \delta } - {1 \over 3} \ln c \right) , \end{aligned}$$

and this is inequality (41). $\square$

Finally, let us estimate the efficiency of method (22) under additional assumption of uniform convexity (28). From the proof of Theorem 3, we know that

$$\begin{aligned} \ln {F_0 \over F_{2m}}\ge & {} {2m \over c^{1/2}} \ln (1+S) \left( {g_{2m} \over g_0} \right) ^{1/(2m)} \; \ge \; {2 m \over c^{1/2}} \ln (1+S) \exp \left( - {1 \over 2m} \ln {g_{0}\over g_{2m}} \right) \\\ge & {} {1 \over c^{1/2}} \ln (1+S) \left( 2m - \ln {g_{0}\over g_{2m}} \right) \; {\mathop {\ge }\limits ^{(35)}} \; {1 \over c^{1/2}} \ln (1+S) \left( 2m - \ln {g_{0}\over \delta } \right) . \end{aligned}$$

On the other hand,

$$\begin{aligned} \ln F_{2m}&{\mathop {\ge }\limits ^{(38)}}&\ln (\kappa m \delta ^{3/2}) + {1 \over 2m} \ln {\delta \over g_{2m}} \; {\mathop {\ge }\limits ^{(20)}} \; \ln (\kappa m \delta ^{3/2}) + {1 \over 2m} \ln {\delta \over g_0} - \ln c. \end{aligned}$$

Thus,

$$\begin{aligned} \ln (cF_0)\ge & {} {2m \over c^{1/2}} \ln (1+S) - {1 \over c^{1/2}} \ln (1+S) \ln {g_{0}\over \delta } + \ln (\kappa m \delta ^{3/2}) + {1 \over 2m} \ln {\delta \over g_0}. \end{aligned}$$

In other words,

$$\begin{aligned} \ln {c F_0 \over \kappa g_0^{3/2}}\ge & {} {2m \over c^{1/2}} \ln (1+S) - {1 \over c^{1/2}} \ln (1+S) \ln {g_{0}\over \delta } + {3 \over 2} \ln {\delta \over g_0} - \ln {1 \over m} + {1 \over 2m} \ln {\delta \over g_0}\\= & {} {2m \over c^{1/2}} \ln (1+S) - \left[ {1 \over 2m} + {1 \over c^{1/2}} \ln (1+S) + {3 \over 2} \right] \ln {g_{0}\over \delta } - \ln {1 \over m}. \end{aligned}$$

Thus, we have proved the following theorem.

### Theorem 6

Let the Hessian be Lipschitz continuous with constant $L_2$. Let $F(\cdot )$ satisfies condition (28). Fix $H \ge L_2$ and some $\delta >0$. Then, the number of iterations of method (22) to reach small norm of the gradient $\Vert F'(x_N) \Vert _{*} \le \delta$ satisfies the following bound:

42 $$\begin{aligned} N&\le {3 c^{1/2} \over 2 \ln (1+S)} \left\{ \ln {c F_0 \over \kappa g_0^{3/2}} + \left[ {1 \over 2m} + {1 \over c^{1/2}} \ln (1+S) + {3 \over 2} \right] \ln {g_{0}\over \delta } \right\} \nonumber \\&{\mathop {\le }\limits ^{(29)}} {3 c^{1/2} \over 2 \ln (1+S)} \ln { 2 c \over 3 \kappa \sqrt{\sigma _3}} + 3 \left[ \frac{1}{2} + {c^{1/2} \over \ln (1+S)} \right] \ln {g_{0}\over \delta }. \end{aligned}$$

## Adaptive search procedure

The main advantage of the method (22) consists in its easy implementation. Indeed, in the case $\psi (\cdot ) \equiv 0$ with $\textrm{dom}\,\psi = {\mathbb {E}}$, the iteration (11) is reduced mainly to matrix inversion, the very standard operation of Linear Algebra, which is available in the majority of software packages. However, for the better performance of this scheme, it is necessary to apply a dynamic strategy for updating the step-size coefficient *H*. Let us show how this can be done.43For the initialization, we need an initial guess $H_0$ for the regularization parameter, which can be an *arbitrary* sufficiently small number.

Note that this scheme does not depend on any particular value of the Lipschitz constant. By definitions of the updates and from inequality (10), we conclude that inequalities $H_0 \le H_k \le L_2$ and $2^{i_k} H_k \le 2L_2$ imply $H_{k+1} \le L_2$. Thus,

44 $$\begin{aligned} H_0 \, \le \, H_k \; \le \; L_2, \qquad 2^{i_k} H_k \; \le \; 2L_2, \qquad k \ge 0. \end{aligned}$$

Hence, from Theorem 1, we have the following progress established for each iteration $k \ge 0$:

$$\begin{aligned} F(x_k) - F(x_{k + 1})\ge & {} \frac{1}{2c_0^2} \sqrt{\frac{3}{2L_2}} \cdot \frac{\Vert F'(x_{k + 1}\Vert _*^2}{\Vert F'(x_k)\Vert _*^{1/2}}, \end{aligned}$$

where

$$\begin{aligned} c_0 {\mathop {=}\limits ^{\textrm{def}}}\sigma ^{-1} + \frac{3L_2}{2H_0}. \end{aligned}$$

Repeating the reasoning of Theorem 2, we obtain the following complexity result.

### Theorem 7

Let the Hessian be Lipschitz continuous with constant $L_2$. Let $F(x_k) - F^* \ge \epsilon$ for some iteration $k \ge 0$ of method (43). Then,

$$\begin{aligned} k\le & {} 4c_0^2 \sqrt{ \frac{2L_2 D^3}{3\epsilon } } + \ln \frac{(F(x_0) - F^{*})\Vert F'(x_0)\Vert _*^{1/2} D^{1/2}}{\epsilon ^{3/2}}. \end{aligned}$$

Note that some scaling of the domain or the target objective may affect the fixed choice of regularization parameter in the basic scheme (22). At the same time, we expect the adaptive method (43) to be robust with respect to these changes.

## Acceleration

Let us present a conceptual acceleration of our method, that is based on the contracting proximal iterations [7].

First, we fix an auxiliary prox-function $\phi (\cdot )$ that we assume to be uniformly convex of degree three with respect to the initial norm:

45 $$\begin{aligned} \beta _{\phi }(x, y)&{=}&\phi (y) {-} \phi (x) {-} \langle \nabla \phi (x), y {-} x \rangle \;\; \ge \;\; \frac{1}{3}\Vert y {-} x\Vert ^3, \quad \forall x, y \in \textrm{dom}\,\psi . \nonumber \\ \end{aligned}$$

At each iteration $k \ge 0$ of the accelerated scheme, we form the following functions:

$$\begin{aligned} g_{k + 1}(x)&{\mathop {=}\limits ^{\textrm{def}}}B_{k + 1} f\Bigl ( \frac{b_{k + 1} x + B_k x_k}{B_{k + 1}} \Bigr ), \\ h_{k + 1}(x)&{\mathop {=}\limits ^{\textrm{def}}}g_{k + 1}(x) + b_{k + 1} \psi (x) + \beta _{\phi }(v_k; x), \end{aligned}$$

where $\{ b_k \}_{k \ge 1}$ is a sequence of positive numbers, $B_k {\mathop {=}\limits ^{\textrm{def}}}\sum \nolimits _{i = 1}^k b_i$, $B_0 {\mathop {=}\limits ^{\textrm{def}}}0$, and

$$\begin{aligned} \{ x_k \}_{k \ge 0}, \quad \{ v_k \}_{k \ge 0}, \quad x_0 = v_0, \end{aligned}$$

are sequences of trial points that belong to $\textrm{dom}\,\psi$.

Note that the derivatives of $g_{k + 1}( \cdot )$ and $f(\cdot )$ are related as follows:

$$\begin{aligned} D^3 g_{k + 1}(x)\equiv & {} \frac{b_{k + 1}^3}{B_{k + 1}^2} D^3 f\Bigl ( \frac{b_{k + 1} x + B_k x_k}{B_{k + 1}} \Bigr ). \end{aligned}$$

For simplicity of the presentation, we assume that *f* is three times differentiable on the open set containing $\textrm{dom}\,\psi$. Let us choose

$$\begin{aligned} b_{k}:= & {} \frac{k^2}{9 L_2(f)}. \end{aligned}$$

Then, $B_k = \frac{1}{9 L_2(f)} \sum \limits _{i = 1}^k i^2 \ge \frac{k^3}{27 L_2(f)}$. Therefore, for any $h \in {\mathbb {E}}$:

$$\begin{aligned} |D^3 g_{k + 1}(x)[h]^3|\le & {} \frac{1}{L_2(f)}\left| D^3 f\Bigl ( \frac{b_{k + 1} x + B_k x_k}{B_{k + 1}} \Bigr )\right| \;\; \le \;\; \Vert h\Vert ^3, \end{aligned}$$

thus $L_2(g_{k + 1}) = 1$, and we can minimize objective $h_{k + 1}$ very efficiently by using our method (22). Namely, in order to find a point *v* with a small norm of a subgradient:

$$\begin{aligned} \Vert g \Vert _{*}\le & {} \delta , \qquad g \in \partial h_{k + 1}(v), \end{aligned}$$

the method needs to do no more than

$$\begin{aligned} N\overset{(41)}{\le } & {} {\tilde{O}}\left( \ln \frac{1}{\delta } \right) \end{aligned}$$

steps, where ${\tilde{O}}(\cdot )$ hides absolute constants and logarithmic factors that depends on the initial residual and subgradient norm.

Let us write down the accelerated algorithm.46Applying directly Theorem 3.2 and the corresponding Corollary 3.3 from [7], we get the following complexity bound.

### Theorem 8

Let the Hessian be Lipschitz continuous with constant $L_2(f)$. Let us set $\delta = \frac{1}{2 \cdot 3^{7/3}} \cdot \bigl (\frac{\epsilon }{L_2(f)}\bigr )^{2/3}$ in method (46), and let

$$\begin{aligned} k= & {} \Bigl \lceil \bigl ( 2 \cdot 3^3 \bigr )^{1/2} \cdot \Bigl ( \frac{L_2(f) \beta _\psi (x_0; x^{*})}{\epsilon } \Bigr )^{1/3} \Bigr \rceil . \end{aligned}$$

Then, $$F(x_k) - F^{*} \le \epsilon . $$ $$\square $$

## Experiments

In this section, let us present computational results for solving the unconstrained minimization problem,

$$\begin{aligned} \min \limits _{x \in {\mathbb {R}}^n} f(x), \end{aligned}$$

with objective that is a smooth convex approximation of pointwise maximum:

$$\begin{aligned} f(x):= & {} \mu \log \biggl ( \sum \limits _{i = 1}^m \exp \Bigl (\frac{\langle a_i, x\rangle - b_i}{\mu }\Bigr ) \biggr ) \;\;\; \approx \;\;\; \max \limits _{i = 1}^m \bigl [ \langle a_i, x \rangle - b_i \bigr ]. \end{aligned}$$

The problems of this type are important in applications with *minimax strategies for matrix games* and $\ell _{\infty }$-*regression* [17].

The vectors $\{ a_i \in {\mathbb {R}}^n \}_{i=1}^m$ and numbers $\{ b_i \in {\mathbb {R}}\}_{i = 1}^m$ are given data, while $\mu > 0$ is a fixed parameter of smoothing.

Let us fix matrix $B:= \sum _{i = 1}^m a_i a_i^T$, which we assume to be positive definite (otherwise, it is possible to reduce the dimensionality of the initial problem), and we use the following Euclidean norms:

$$\begin{aligned} \Vert x\Vert:= & {} \langle Bx, x \rangle ^{1/2}, \qquad \Vert g\Vert _* \;\;:= \;\; \langle g, B^{-1}g\rangle ^{1/2}, \end{aligned}$$

respectively for the variables and for the gradients. We also know the corresponding Lipschitz constant for the Hessian, that is (see, e.g. Example 1.3.6 in [6])

47 $$\begin{aligned} L_2:= & {} \frac{2}{\mu ^2}. \end{aligned}$$

To generate the data, we sample random elements $\{\bar{a}_i \in {\mathbb {R}}^n, b_i \in {\mathbb {R}}\}_{i = 1}^m$ from the uniform distribution on $[-1, 1]$, and form an auxiliary function

$$\begin{aligned} \bar{f}(x):= & {} \mu \log \left( \sum \limits _{i = 1}^m \exp \left( \frac{\langle \bar{a}_i, x\rangle - b_i}{\mu } \right) \right) . \end{aligned}$$

Then, we set

$$\begin{aligned} a_i:= & {} \bar{a}_i - \nabla \bar{f}(0), \qquad 1 \le i \le m. \end{aligned}$$

Thus we ensure to have the optimum at the origin, since $\nabla f(0) = 0$. We start the methods from $x_0:= (1, 1, \ldots , 1)$.

We study the performance of the Newton method with Gradient regularization and with Cubic regularization [19] on this problem. Also, we compare our accelerated scheme (46) with the basic methods.

We use the following scaling function for this problem, as in Example 1:

$$\begin{aligned} d(x)= & {} \frac{1}{2}\Vert x\Vert ^2 \; = \; \frac{1}{2}\langle Bx, x \rangle . \end{aligned}$$

The subproblem in our methods is solved exactly by using the standard matrix inversion. For the Cubic Newton, one need to find a root of a one-dimensional nonlinear equation at each step (see Section 5 in [19]). To solve it, we apply the classical univariate Newton method and use the value $\epsilon = 10^{-8}$ as a target tolerance in terms of the function value.

Regularization parameter is fixed according to the theory (47). The results are shown in Fig. . We see that both algorithms show reasonably good performance, which is better than the theoretical prediction of the global behaviour. The Newton method with Gradient regularization possesses the best convergence rate. Accelerated scheme has an improvement in the rate in the beginning, but the basic methods are better for the higher level of the accuracy due to their superlinear local convergence.

**Fig. 1:**
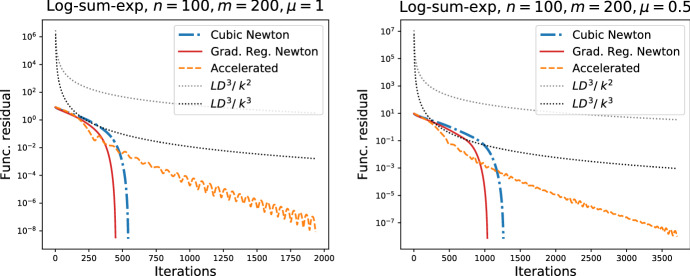
Newton methods with Cubic and with Gradient regularization, and the accelerated scheme. Lipschitz constant is fixed

In the following experiment, we compare the uses of the fixed Lipschitz constant with the adaptive search procedure for our method. The results are shown in Fig. . We see that the adaptive methods show the best performance. At the same time, iterations of the Gradient regularization are much cheaper which results in better computational time.

**Fig. 2:**
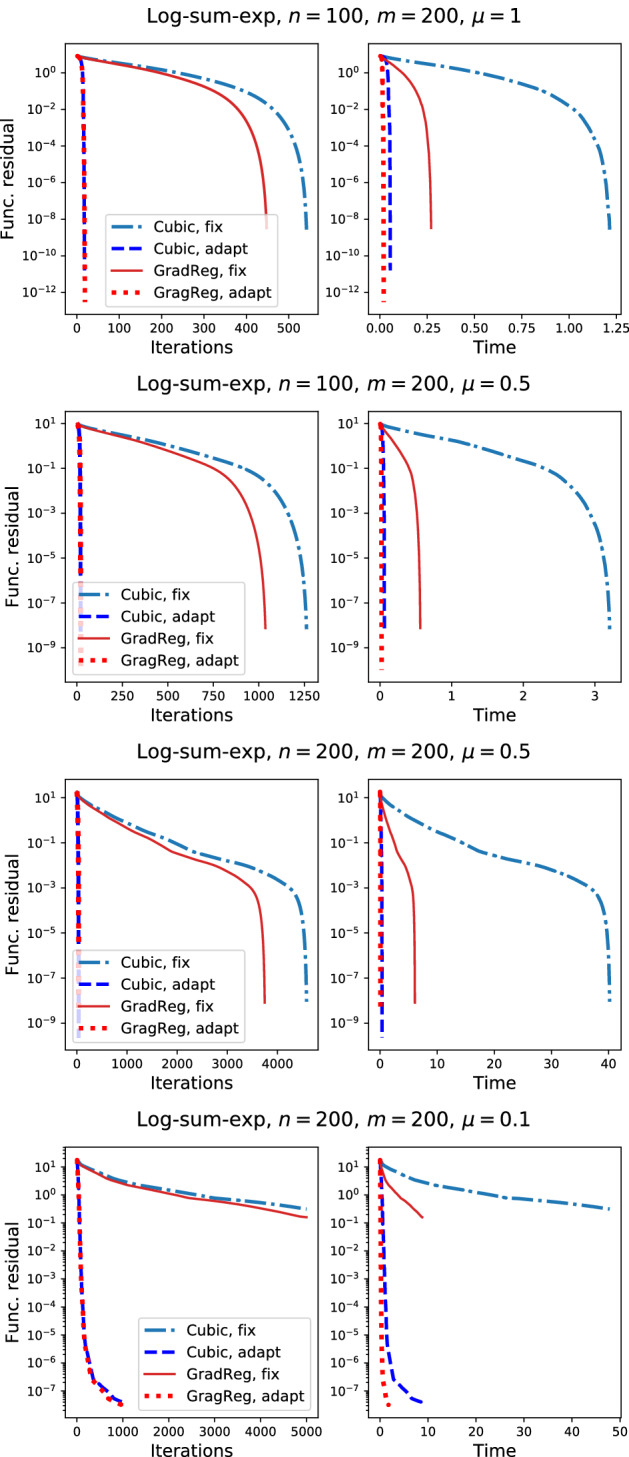
The effect of using adaptive line search in the regularized Newton methods

Finally, we compare our approach with iterations of the *damped* Newton method with line search. For this problem, the Hessian is often degenerate, thus we use a small perturbation to correct the matrix. Namely, we consider the following iterations:

$$\begin{aligned} x_{k + 1}= & {} x_k - \alpha _k \Bigl ( \nabla ^2 f(x_k) + \tau B \Bigr )^{-1} \nabla f(x_k), \quad k \ge 0, \end{aligned}$$

where $\tau$ is a fixed small parameter (we set $\tau = 10^{-6}$ which was tuned to have the best performance), and $\alpha _k$ is chosen by the standard backtracking line search to satisfy the following condition:

$$\begin{aligned} f(x_k) - f(x_{k + 1})\ge & {} \frac{\alpha _k}{2}\Vert \nabla f(x_k) \Vert _{*}^2. \end{aligned}$$

The results are presented in Fig. . We see that the damped Newton method is sensitive to the choice of perturbation parameter $\tau$, while the method with Gradient regularization shows the most robust and efficient performance for all problem instances.

**Fig. 3:**
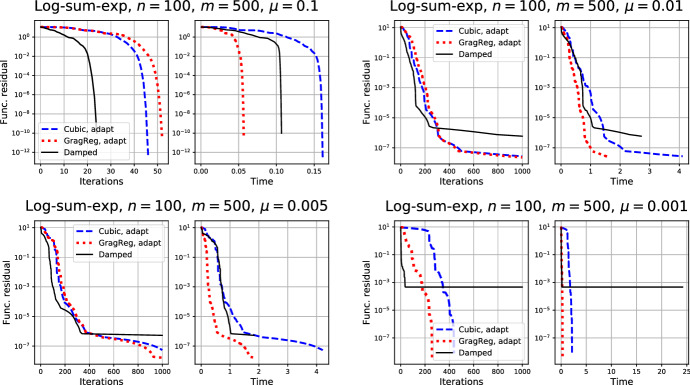
Comparison of the regularized Newton methods with the damped Newton algorithm

## Discussion

In this paper, we have analyzed the global behaviour of the Newton method with a general Bregman regularizer, whose regularization parameter is chosen to be proportional to the square root of the current gradient norm.

We demonstrated that our scheme works with the composite form of the convex optimization problem. For the Euclidean norms, this approach can be seen as a *relaxation* of the Cubically regularized Newton method, achieving the same global convergence rates.

A significant advantage of the gradient regularization scheme is a simpler structure of the subproblem, which does not need auxiliary one-dimensional minimizations that are required in the cubic regularization. As a consequence, the subproblem becomes suitable for the large-scale case as for employing the Conjugate Gradient methods.

It is a favorable feature of our methods that regularization parameter always depends on the *current* iterate only. Therefore, it seems to be convenient for the use in stochastic optimization. We believe that this property could fit well with the broad family of stochastic second-order methods based on the Cubic regularization (see [4, 12, 14]). We keep the development of such schemes for further investigation.

Another important direction is an extension of our results to nonconvex optimization problems. It seems to be a challenging question since our current analysis heavily relies on positive semidefiniteness of the Hessian. It is needed to ensure a bound for the step length (see Lemma 1). Therefore, to tackle nonconvex problems, some modifications of our analysis have to be made.

## Footnotes

1 When this paper was already finished, we discovered that this idea was recently proposed by K. Mishchenko [16] for solving unconstrained minimization problem with smooth objective. As compared to his work, our main advances consist in the usage of Bregman distances, composite form of optimization problem, linear rate of convergence for uniformly convex functions, and developments of accelerated variant of the method.
